# Antibiotic resistance: knowledge, attitudes, and prescribing behaviors among dental students: a cross-sectional study

**DOI:** 10.3389/froh.2025.1638336

**Published:** 2025-10-24

**Authors:** Mayar Danadneh, Raghad Saleh, Aya Abu Kwaik, Razan Abu Lafi, Amin Yasin, Ghaida Saafeen, Mazen Alwahidi, Wendy Thompson, Elham Kateeb

**Affiliations:** 1 Oral Health Research and Promotion Unit, Al-Quds University, Jerusalem, Palestine; 2 Stem Cell and Regenerative Therapies, Faculty of Life Sciences and Medicine, King’s College London, London, United Kingdom; 3 MSc Oral and Maxillofacial Surgery, Eastman Dental Institute, University College London, London, United Kingdom; 4 School of Dentistry, Al Azhar University, Gaza, Palestine; 5 Division of Dentistry, University of Manchester, Manchester, United Kingdom

**Keywords:** antibiotic resistance, dental students, prescribing practices, Palestine, antibiotic stewardship, education

## Abstract

**Background:**

Antimicrobial resistance (AMR) is a global crisis exacerbated by inappropriate antibiotic use, including in dentistry. This study evaluates the knowledge, attitudes, and prescribing behaviours of Palestinian dental students to identify gaps and inform educational interventions.

**Methods:**

A cross-sectional study was conducted among 4th, 5th, and internship-year dental students from four Palestinian dental schools. A validated online survey assessed knowledge, attitudes, confidence, and prescribing behaviours. Statistical analyses, including chi-square tests and Pearson correlations, examined associations between knowledge, confidence, and prescribing practices.

**Results:**

Among 586 participants (38% response rate), 72.2% recognized antibiotic misuse as a resistance driver, yet only 40.3% felt confident in prescribing decisions. Inappropriate prescribing was reported, with 13.3% prescribing antibiotics daily due to diagnostic uncertainty (*χ*^2^ = 504.414, *p* = 0.000) and 12.1% due to patient expectations (*χ*^2^ = 670.491, *p* = 0.000). Higher perceived knowledge correlated with reduced prescribing frequency (*χ*^2^ = 82.650, *p* = 0.000), and confidence in guidelines was positively associated with responsible prescribing (*χ*^2^ = 79.656, *p* = 0.000). Access to prescribing guidelines significantly reduced antibiotic misuse (*χ*^2^ = 106.441, *p* = 0.000). Interns prescribed antibiotics more frequently than juniors (*χ*^2^ = 55.618, *p* = 0.000).

**Conclusions:**

Significant gaps in knowledge, confidence, and prescribing behaviours among Palestinian dental students highlight the urgent need for enhanced antibiotic stewardship education. Implementing targeted interventions, standardized guidelines, and improved access to prescribing resources is essential to promote responsible antibiotic use and combat AMR in dentistry.

## Introduction

Despite significant advances in pharmaceuticals and their widespread use, the global burden of diseases continues to increase. Factors such as rapid globalization and economic development have significantly contributed to the rising incidence of illnesses worldwide. Medications play a crucial role in maintaining health, reducing morbidity, managing chronic illnesses, and curing diseases. Effective communication of medication plans through prescriptions is essential for the safe and efficient delivery of medications to patients. However, prescribing drugs is a complex task that requires both theoretical knowledge and practical experience. Recently, there has been growing concern over the misuse of medications, including self-medication by patients and inappropriate prescribing practices by healthcare professionals (1).

Antimicrobial Resistance (AMR) is a critical global health challenge that complicates the treatment of infectious diseases and undermines the effectiveness of established therapies (1). While resistance is a natural evolutionary process in microorganisms, substantial evidence indicates that its acceleration is largely driven by the overuse and misuse of antimicrobials. According to the Centres for Disease Control and Prevention (CDC), AMR occurs when drugs fail to eliminate the organisms they are designed to target, allowing these pathogens to survive, grow, and spread. This presents a significant global health threat, as it makes the treatment of common infections increasingly difficult (1).

The escalating use of antibiotics by healthcare professionals, particularly physicians and dentists (2), has led to their misapplication and overuse, significantly contributing to the rise in antibiotic resistance (3, 4). As a result, AMR has become a major concern across all healthcare sectors, including dentistry, highlighting the urgent need to optimise prescribing practices, especially in dental care.

Studies have revealed alarming statistics regarding antibiotic prescriptions in dentistry. For example, research in the United Kingdom found that only 19% of antibiotics prescribed for acute dental conditions followed the recommendations of the guidelines (5). Similarly, a study in the United States found inappropriate antibiotic prescriptions for prophylaxis use in more than 80% of the cases (6). Such high rates of inappropriate antibiotic prescribing by dentists underscore the essential role of the dental profession in tackling the rise of bacterial resistance (7).

In dentistry, the most commonly prescribed medications are non-steroidal anti-inflammatory drugs (NSAIDs) and antibiotics (8, 9) However, inappropriate antibiotic prescriptions in dental practice have been linked to serious adverse outcomes, including anaphylaxis, colitis, and the development of drug-resistant infections (10, 11). To help dentists balance the risks of dental infections with the potential harms of antibiotic prescriptions, clinical guidelines have been developed (7, 12).

In Palestine, where dental education and practice face unique challenges due to ongoing severe geopolitical conflict, no studies have yet assessed antibiotic use among dentists (13). However, two recent studies on antibiotic use among primary care providers in Palestine raised concerns about the overuse of these medications (14, 15).

Understanding prescribing practices among future dentists is therefore essential. Palestinian dental students, from juniors to interns, are at the forefront of learning and applying pharmacological knowledge in clinical settings. Typically, they gain clinical experience by treating patients under close supervision in university outpatient clinics. Students are introduced to antibiotic use during pharmacology courses and are expected to apply this knowledge in clinical training under supervision during their 4th, 5th, and internship years, aiming to develop safe prescribing skills based on university-developed guidelines (16, 17).

Despite numerous studies globally examining the antibiotic knowledge and prescribing behaviours of dental practitioners, most have predominantly focused on practising dentists (18), with limited attention given to the knowledge and attitudes of dental students. Therefore, this study aimed to evaluate the knowledge, attitudes, and prescribing behaviors of Palestinian dental students regarding antibiotic use. Given the unique challenges in Palestinian dental education and healthcare, including the absence of studies on antibiotic prescribing among dentists in Palestine, this study sought to fill this gap by exploring antibiotic stewardship at the educational level. It aims to identify key factors influencing prescribing decisions among dental students, with a focus on the development of safe and evidence-based antibiotic prescribing practices in clinical settings.

## Study-specific objectives

1. Assess the actual knowledge of Palestinian dental students about antibiotic use, including understanding antibiotic resistance, appropriate antibiotic selection, and common misconceptions such as the use of antibiotics for viral infections.
2. Examine the attitudes of dental students toward antibiotic prescribing, focusing on their perceptions of their role in controlling antibiotic resistance, the connection between prescribing practices and resistance, and their confidence in making decisions related to antibiotic use.
3. Evaluate the self-reported prescribing behaviors of dental students in clinical practice, identifying practices deemed as “good” or “bad/inappropriate” according to established guidelines for antimicrobial resistance (AMR).
4. Investigate students' perceived access to antibiotic prescription guidelines and materials, and their opportunities to provide advice on prudent antibiotic use to patients, identifying barriers or challenges to effective antibiotic stewardship.
5. Identify the relationship between dental students' knowledge levels, attitudes, confidence in prescribing, and the accessibility of resources, aiming to understand how these factors collectively influence their antibiotic prescribing behaviors.
6. Examine the differences in “Actual Knowledge” scores across demographic variables (e.g., gender, year of study, family background in the medical field).
7. Explore correlations between “Actual Knowledge” and Perceived Knowledge, Confidence in prescribing antibiotics, and attitudes toward antibiotic resistance.
8. Investigate the relationship between “Confidence in prescribing antibiotics” and access to guidelines, educational materials, and opportunities for advice.
9. To evaluate the factors influencing antibiotic prescribing frequency among dental students, including the association with “Year of Study,” the impact of knowledge-related variables (such as knowledge of antibiotic resistance and confidence in prescribing decisions), the effect of access to resources (guidelines, materials, and opportunities for advice), and the role of attitudes and behaviours related to antibiotic resistance.
10. Assess how inappropriate prescribing practices (e.g., uncertainty about infection diagnosis, early discontinuation of prescriptions) influence antibiotic prescribing frequency.

The findings of this study will inform dental educators, professional associations, and health ministries in developing dental antibiotic stewardship interventions, including educational programs and standardised guidelines for Palestine. By promoting appropriate prescribing practices, the Palestinian dental community can contribute to global efforts to preserve the efficacy of antimicrobial agents and ensure optimal patient outcomes.

## Materials and methods

### Study design & setting

A cross-sectional study was conducted among dental students in their fourth, fifth, and internship years of study at the four Palestinian dental schools in the West Bank, Gaza, and East Jerusalem from January 2023–May 2023.

### Sampling & data collection

The questionnaire targeted all clinical students (4th, 5th, and Internship years) across the four accredited dental schools in the Palestinian Territories (*N* = 2,067): Al-Quds University (East Jerusalem), Arab American University (Jenin), Palestine University (Gaza), and Al-Azhar University (Gaza). At the time of the survey, the number of clinical students in each university was as follows: Al-Quds University (507 students), Arab American University (930 students), Al-Azhar University (360 students), and Palestine University (270 students). Data collection was conducted using Google Forms, which were distributed via the Messenger app and the official Facebook groups of the dental schools—both standard communication channels in these institutions. The survey introduction provided participants with detailed information about the study's objectives, emphasised the voluntary nature of participation, and explained the measures taken to ensure confidentiality and anonymity. A sample size calculation was performed using the Raosoft online calculator, with a 95% confidence level and a 5% margin of error, resulting in a minimum required sample size of (*n* = 325).

### Recruitment process

A student representative was selected from each participating university to facilitate the recruitment process. These representatives were responsible for disseminating the survey and encouraging participation among their peers. This strategy aimed to foster commitment and ensure balanced representation from all participating institutions.

### Instrument development

The study's self-administered questionnaire was adapted from a previously validated instrument by Karasneh et al. originally used among physicians and dentists in Jordan (16). To ensure its suitability for the target population, a pilot test was conducted with ten dental students from Al-Quds University in their fourth, fifth, and internship years, reflecting the characteristics of the main study sample. The pilot aimed to refine the questionnaire for clarity and relevance. On average, participants took about 7 min to complete the survey. The full questionnaire is provided in Tables 2–7, 8B.

The study questionnaire consisted of 40 multiple-choice items, divided into three sections. The first section collected socio-demographic information (Table 1), while the second and third sections focused on respondents' knowledge (Table 2) and attitudes towards antibiotic use and resistance (Tables 3, 4), as well as their prescribing practices (Tables 6, 7).

**Table 1:** Respondents' sociodemographic characteristics.

Variable	Frequency (*n*)	Percent (%)
Gender		
Male	213	36.3
Female	373	63.7
University		
Al-Quds University	250	42.7
Palestine University	62	10.6
Arab American University	163	27.8
Al-Azhar University	111	18.9
Year of Study		
4th	216	36.9
5th	186	31.7
Interns	184	31.4
Having a family member/relative working in the medical field		
Yes	347	59.2
No	239	40.8

**Table 2:** Respondents' actual & perceived knowledge.

Questions	Frequency (*n*)	Percent (%)
Actual Knowledge		
Antibiotics are effective against viruses		
True	161	27.5
False	384	65.5
Unsure	41	7.0
Antibiotics are effective against cold infections		
True	212	36.2
False	279	47.6
Unsure	95	16.2
Unnecessary use of antibiotics make them become ineffective		
True	423	72.2
False	122	20.8
Unsure	41	7.0
Taking antibiotics has associated side effects or risks such as diarrhoea, colitis, allergies		
True	415	70.8
False	96	16.4
Unsure	75	12.8
Every person treated with antibiotics is at an increased risk of antibiotic-resistant infection		
True	401	68.4
False	116	19.8
Unsure	69	11.8
Antibiotic-resistant bacteria can spread from person to person		
True	252	43.0
False	201	34.3
Unsure	133	22.7
Healthy people can carry antibiotic-resistant bacteria		
True	319	54.4
False	133	22.7
Unsure	134	22.9
Perceived Knowledge		
I know what antibiotic resistance is		
Strongly disagree/Disagree	174	29.7
Undecided	70	11.9
Strongly Agree/Agree	338	57.7
I do not understand the question	4	.7
I have sufficient knowledge about how to use antibiotics appropriately for my current training		
Strongly disagree/Disagree	195	33.2
Undecided	128	21.8
Strongly Agree/Agree	249	42.5
I do not understand the question	8	1.4
I know what information to give to individuals about prudent use of antibiotics and antibiotic resistance		
Strongly disagree/Disagree	191	32.6
Undecided	128	21.8
Strongly Agree/Agree	252	43
I do not understand the question	14	2.4

**Table 3:** Descriptive statistics of “confidence scale” statements.

Questions	Frequency (*n*)	Percent (%)
I am confident in making antibiotic-prescribing decisions		
Strongly disagree/Disagree	190	32.4
Undecided	142	24.2
Strongly Agree/Agree	236	40.3
I do not understand the question	13	2.2
Not Applicable	5	.9
I have confidence in the antibiotic guidelines available to me		
Strongly disagree/Disagree	192	32.8
Undecided	152	25.9
Strongly Agree/Agree	235	40.1
I do not understand the question	5	.9
Not Applicable	2	.3

**Table 4:** Descriptive statistics of “attitude towards students' role in fighting antibiotic resistance” statements.

Questions	Frequency (*n*)	Percent (%)
I have a key role in helping control antibiotic resistance		
Strongly disagree/Disagree	190	32.4
Undecided	142	24.2
Strongly Agree/Agree	239	40.8
I do not understand the question	11	1.9
Not Applicable	4	.7
I know there is a connection between my prescribing of antibiotics and the emergence and spread of antibiotic-resistant bacteria		
Strongly disagree/Disagree	164	28
Undecided	94	16.0
Strongly Agree/Agree	325	55.4
I do not understand the question	2	.3
Not Applicable	1	.2

**Table 5:** The frequency of antibiotics prescription.

Questions	Frequency (*n*)	Percent (%)
How often do you prescribe antibiotics		
Everyday	91	15.5
Weekly	136	23.2
Monthly	156	26.6
Quarterly	100	17.1
Yearly	103	17.6
How often did you prescribe antibiotics during the last one week?		
More than once a day	63	10.8
Once a Day	110	18.8
More than once a week	71	12.1
Once a week	131	22.4
Rarely	74	12.6
Never	83	14.2
I do not remember	21	3.6
Not Applicable	33	5.6

**Table 6:** Descriptive statistics of practices related to antibiotic prescriptions.

Questions	Frequency (*n*)	Percent (%)
How often did you give out resources (e.g., leaflets or pamphlets) on prudent antibiotic use or management of infections to individuals during the last one week?		
Once a day	70	11.9
More than once a day	84	14.3
Once a week	115	19.6
More than once a week	52	8.9
Rarely	94	16.0
Never	107	18.3
I do not remember	28	4.8
Not Applicable	36	6.1
How often did you give out advice related to prudent antibiotic use or management of infections to an individual during the last one week?		
Once a day	73	12.5
More than once a day	93	15.9
Once a week	110	18.8
More than once a week	71	12.1
Rarely	107	18.3
Never	78	13.3
I do not remember	29	4.9
Not Applicable	25	4.3
I consider antibiotic resistance when treating a patient		
Strongly disagree/Disagree	180	30.7
Undecided	126	21.5
Strongly Agree/Agree	265	45.3
I do not understand the question	12	2.0
Not Applicable	3	0.5
I feel supported to not prescribe antibiotics when they are not necessary		
Strongly disagree/Disagree	181	30.9
Undecided	112	19.1
Strongly Agree/Agree	272	46.4
I do not understand the question	16	2.7
Not Applicable	5	.9

**Table 7:** The frequency and percentages of bad/inappropriate practices related to antibiotic prescription.

Questions	Frequency (*n*)	Percent (%)
How often would you have preferred not to prescribe an antibiotic but were not ableo during the last one week?		
Once a day	76	13.0
More than once a day	86	14.7
Once a week	114	19.5
More than once a week	76	13.0
Rarely	89	15.2
Never	89	15.2
I do not remember	28	4.8
Not Applicable	28	4.8
How often did the fear of patient deterioration or fear of complications lead you to prescribe antibiotics during the last one week?		
Once a day	69	11.8
More than once a day	93	15.9
Once a week	118	20.1
More than once a week	61	10.4
Rarely	102	17.4
Never	95	16.2
I do not remember	23	3.9
Not Applicable	25	4.3
How often did you prescribe antibiotics because it took less time than to explain the reason why they were not indicated during the last one week?		
Once a day	69	11.8
More than once a day	83	14.2
Once a week	98	16.7
More than once a week	64	10.9
Rarely	92	15.7
Never	120	20.5
I do not remember	28	4.8
Not Applicable	32	5.5
How often did you prescribe antibiotics in situations in which it is impossible for you to conduct a follow-up of the patient during the last one week?		
Once a day	71	12.1
More than once a day	76	13.0
Once a week	118	20.1
More than once a week	57	9.7
Rarely	93	15.9
Never	107	18.3
I do not remember	31	5.3
Not Applicable	33	5.6
How often did you prescribe an antibiotic because you were uncertain about the diagnosis of infection during the last one week?		
Once a day	78	13.3
More than once a day	65	11.1
Once a week	108	18.4
More than once a week	68	11.6
Rarely	86	14.7
Never	117	20.0
I do not remember	25	4.3
Not Applicable	39	6.7
How often did you prescribe an antibiotic to maintain the relationship with the patient during the last one week?		
Once a day	71	12.1
More than once a day	76	13.0
Once a week	95	16.2
More than once a week	64	10.9
Rarely	86	14.7
Never	128	21.8
I do not remember	25	4.3
Not Applicable	41	7.0
How often did you stop an antibiotic prescription earlier than the prescribed course length during the last one week?		
Once a day	69	11.8
More than once a day	77	13.1
Once a week	104	17.7
More than once a week	56	9.6
Rarely	89	15.2
Never	125	21.3
I do not remember	28	4.8
Not Applicable	38	6.5
How often did you prescribe a shorter course of treatment as compared to available guidelines during the last one week?		
Once a day	67	11.4
More than once a day	70	11.9
Once a week	103	17.6
More than once a week	67	11.4
Rarely	85	14.5
Never	122	20.8
I do not remember	31	5.3
Not Applicable	41	7.0
How often did you discontinue early (within three days after initiation) a treatment because the bacterial infection was not likely after all during the last one week?		
Once a day	79	13.5
More than once a day	68	11.6
Once a week	91	15.5
More than once a week	70	11.9
Rarely	87	14.8
Never	117	20.0
I do not remember	29	4.9
Not Applicable	45	7.7

Confidence questions were added to assess participants' self-reported confidence in making decisions about antibiotic prescribing and their confidence in the available antibiotic guidelines. The questions were: “I am confident in making antibiotic prescribing decisions” and “I have confidence in the antibiotic guidelines available to me”.

Responses to the questions related to perceived knowledge, attitude and confidence, were measured using a 7-point Likert scale, where 1 represented “Strongly disagree”, 2 “Disagree”, 3 “Undecided”, 4 “Agree”, 5 “Strongly agree”, 6 “I do not understand the question”, and 7 “Not Applicable”. Actual knowledge questions have three possible answers: correct statement, incorrect or unsure from the answer. Practices related to antibiotic prescription were classified as “Good Practices” or “Inappropriate (Bad) Practices” in accordance with established international guidelines on antimicrobial resistance (AMR) (19). The evaluation of antibiotic use comprised six self-reported items designed to assess participants' adherence to evidence-based prescribing standards and their engagement in potentially inappropriate antibiotic practices. Each item was measured using a self-reported scale with response options coded to indicate the frequency of practices: 1 = Never, 2 = Rarely, 3 = Once a week, 4 = More than once a week, 5 = Once a day, 6 = More than once a day, 7 = I do not remember and 8 = Not Applicable (8).

The questionnaire also included questions that assessed Antibiotic Prescription Guidance Accessibility. Three statements were rated using 7-point Likert scale, where 1 represented “Strongly disagree”, 2 “Disagree”, 3 “Undecided”, 4 “Agree”, 5 “Strongly agree”, 6 “I do not understand the question”, and 7 “Not Applicable”.

The main dependent variable in this study, antibiotics prescription frequency, was measured by the question: How often did you prescribe antibiotics during the last one week? And the answers were, 1 = Never, 2 = Rarely, 3 = Once a week, 4 = More than once a week, 5 = Once a day, and 6 = More than once a day.

### Statistical analysis

To further our analysis, four composite variables were constructed: **Levels of Actual Knowledge**, **Perceived Knowledge**, the **Attitude Scale**, and the **Confidence Scale**.

The first composite variable was **“Levels of Actual Knowledge”** included seven questions assessing knowledge about antibiotic use and resistance. These questions were: “Antibiotics are effective against viruses”, “Antibiotics can be used to treat bacterial infections”, “Overuse of antibiotics contributes to resistance”, “Finishing a prescribed antibiotic course is important to prevent resistance”, “It is safe to take leftover antibiotics”, “Antibiotics should be taken for a cold or flu”, and “Using antibiotics when they are not needed accelerates resistance.” Each question had a binary response (1 for correct answer, 0 for incorrect or unsure answers), resulting in a possible score range of 0–7.

The second composite variable, **“Perceived Knowledge”**, comprised three questions: “I know what antibiotic resistance is”, “I have sufficient knowledge about how to use antibiotics appropriately for my current training”, and “I know what information to give individuals about prudent use of antibiotics and antibiotic resistance”. The total possible scores for Perceived Knowledge, using a summation method, range from 3–15, and a Cronbach's Alpha of 0.84, indicating good internal consistency.

The third composite variable, **“Attitude Scale”**, consisted of two items: “I have a key role in helping control antibiotic resistance.” and “There is a connection between my prescribing practices and antibiotic resistance.” With a total score range of 2–10 and a Cronbach's Alpha of 0.74, demonstrating acceptable internal consistency.

The **Confidence Scale** comprised two items: “I am confident in making antibiotic prescribing decisions” and “I have confidence in the antibiotic guidelines available to me”, with a total score range of 8, a minimum score of 2, and a maximum score of 10 Cronbach's Alpha of confidence composite scale was 0.874.

In addition to the four composite variables (Levels of Actual Knowledge, Perceived Knowledge, the Attitude Scale, and the Confidence Scale), a separate composite variable, **“Antibiotic Prescription Guidance Accessibility”** was constructed to assess students' perceived access to antibiotic guidelines and their opportunities to provide advice on prudent antibiotic use. The composite variable consisted of the following statements: “I have easy access to guidelines I need on managing infections.”, “I have easy access to the materials I need to give advice on prudent antibiotic use and antibiotic resistance.”, and “I have good opportunities to provide advice on prudent antibiotic use to individuals”. Higher scores indicated greater perceived opportunities to appropriately prescribe and advise on antibiotic use. Using a summation method, range of 12 with a minimum of 3, a maximum of 15, and a Cronbach's Alpha of 0.87, indicating very good internal consistency.

Statistical analysis was performed using the Statistical Package for the Social Sciences (SPSS) version 23.0. Descriptive statistics were used to summarize frequencies and proportions of responses to key survey items, including knowledge, perceived knowledge, confidence, attitudes, and self-reported practices.

To assess whether the observed distributions of agreement levels (e.g., “strongly agree” to “strongly disagree”) differed significantly from an expected distribution, Chi-square goodness of fit tests were conducted. These tests assumed an equal distribution across response categories as the null hypothesis, allowing for the identification of skewed patterns in participant responses. Additionally, bivariate associations between categorical variables were examined using the Pearson Chi-square test, while differences in continuous variables across categories were analyzed using the *t*-test and ANOVA. The relationship between continuous variables, composite variables, and scales was assessed using Pearson's Correlation test. Relationships between continuous and ordinal variables, as well as between pairs of continuous composite variables, were assessed using Spearman's Rank test when the assumptions of normality and linearity were not met.

In general, the analysis focused on examining factors influencing antibiotic prescribing frequency, including the association with the different study composite variables and some other key variables such as “Year of Study”, the impact of knowledge-related variables (e.g., knowledge of antibiotic resistance and confidence in prescribing decisions), access to resources (e.g., guidelines, materials, and opportunities for advice), and attitudes and behaviours related to antibiotic resistance. Statistical significance was set at *p* < 0.05 for all tests.

## Ethical considerations

The study was approved by the research ethics committee at Al-Quds University, ensuring compliance with all necessary ethical standards. The committee's decision letter was received on December 12, 2022, with reference No. 222/REC/2022.

## Results

### Descriptive statistics

A total of 586 Palestinian dental students participated in the online questionnaire, achieving a response rate of 38%. The majority of respondents were female, representing 63.7% (*n* = 373) of the sample. Regarding academic year distribution, 36.9% were in their 4th year (*n* = 216), 31.7% in their 5th year (*n* = 186), and 31.2% in their internship year (*n* = 184). Additional sociodemographic characteristics are presented in Table 1.

A strong foundational understanding of antibiotic resistance was evident, with 72.2% of students (*n* = 423) correctly identifying that unnecessary use of antibiotics contributes to their ineffectiveness.

In terms of perceived knowledge, 57.7% (*n* = 338) of students agreed or strongly agreed that they understood the concept of antibiotic resistance. The distribution of responses was significantly skewed toward agreement (*χ*^2^ = 271.3, *p* < 0.001), reflecting non-random clustering around higher self-perceived knowledge.

When asked whether their current training provided sufficient knowledge on the appropriate use of antibiotics, only 42.5% (*n* = 294) expressed agreement. This item also showed a significantly non-uniform distribution of responses (*χ*^2^ = 320.9, *p* < 0.001), indicating a polarized view among students regarding the adequacy of their training.

Confidence in antibiotic prescribing emerged as a concern. Only 40.3% (*n* = 236) of students reported feeling confident in their ability to make prescribing decisions, and a similar proportion (40.1%, *n* = 235) expressed confidence in the available guidelines. Both items demonstrated statistically significant deviations from a uniform distribution (*χ*^2^ = 303.0 and *χ*^2^ = 271.3, respectively; both *p* < 0.001), suggesting a concentration of responses in the lower-to-middle confidence range.

Access to necessary resources for prudent antibiotic prescribing was also limited. Just 37.7% of students agreed or strongly agreed that they had easy access to relevant guidelines and educational materials. The distribution of responses significantly departed from uniformity (*χ*^2^ = 301.2, *p* < 0.001), underscoring constraints in resource accessibility across the sample.

Students' attitudes toward their professional role in combating antibiotic resistance revealed divided opinions. While 40.8% perceived themselves as having a key role in antibiotic stewardship, 32.4% disagreed or strongly disagreed, and 24.2% remained undecided. This wide spread of responses was statistically significant (*χ*^2^ = 303.0, *p* < 0.001), highlighting variability in students' sense of responsibility.

Finally, when students were asked whether they recognized the link between prescribing antibiotics and the development of resistant bacteria, 55.4% agreed or strongly agreed, 30% disagreed or strongly disagreed, and 16% were undecided. The distribution was significantly skewed toward agreement (*χ*^2^ = 410.5, *p* < 0.001), indicating widespread awareness of this important public health issue.

Overall, the sample reported an average score of 4.22 ± 1.58 out of 7 on the “Actual Knowledge” composite variable and 9.45 ± 3.41 out of 15 on the “Perceived Knowledge” composite variable. Scores on the “Attitude Scale” ranged from 2–10, with a mean of 6.36 ± 2.24, while the “Confidence Scale” had a mean score of 6.07 ± 2.31 out of 10. Additionally, “Antibiotic Prescription Guidance Accessibility” scale ranged from 3–15 with a mean score of 9.12 ± 3.59.

An analysis of the respondents' antibiotic prescription patterns revealed that a significant proportion prescribed antibiotics regularly: 15.5% reported prescribing them daily, 23.2% weekly, and 26.6% monthly. When asked specifically about their prescribing practices in the past week, 18.8% indicated they had prescribed antibiotics once a day, while 22.4% reported doing so once a week.

Our findings highlighted several practices related to prescribing antibiotics to patients. Among the respondents, 19.6% reported providing resources (e.g., leaflets or pamphlets) on prudent antibiotic use or infection management to individuals once a week, while 18.3% indicated they never distributed such materials. Regarding offering advice on prudent antibiotic use or infection management, 12.5% provided advice once a day, and 18.8% did so once a week.

Additionally, 45.3% of participants “agreed” or “strongly agreed” that they consider antibiotic resistance when treating patients, whereas 30.7% did not. Moreover, 46.4% of respondents felt supported in avoiding unnecessary antibiotic prescriptions, while 30.9% felt unsupported in this regard.

Our results also revealed several prevalent inappropriate practices related to antibiotic prescribing. In the past week, 11.8% of respondents reported prescribing antibiotics once a day out of fear of potential deterioration or complications, while 15.9% did so more than once daily.

Another concerning behaviour was prescribing antibiotics because it required less time than explaining why they were not indicated. Among respondents, 11.8% admitted to doing this once a day, and 14.2% did so more than once daily.

In scenarios where follow-up with patients was impossible, 12.1% of respondents prescribed antibiotics once a day, 13.0% more than once daily, 20.1% once a week, and 9.7% more than once a week.

Uncertainty about infection diagnosis also influenced students' prescribing practices: 13.3% of respondents did this once a day, 11.1% more than once daily, 18.4% once a week, and 11.6% more than once a week.

A notable proportion of respondents reported prematurely stopping antibiotic prescriptions within the last week. Specifically, 11.8% stopped prescriptions once a day, 13.1% more than once daily, 17.7% once a week, and 9.6% more than once a week. Similarly, prescribing shorter courses of treatment than recommended by guidelines was reported by 11.4% of respondents once a day, 11.9% more than once daily, 17.6% once a week, and 11.4% more than once a week.

Finally, the discontinuation of antibiotic treatment within three days after initiation because bacterial infection was deemed unlikely was reported by 13.5% of respondents once a day, 11.6% more than once daily, 15.5% once a week, and 11.9% more than once a week.

In addition to exploring knowledge, attitudes, practices, and confidence related to antibiotic prescribing, this study assessed **policy-related questions** to understand students' perspectives on effective strategies for combating antibiotic resistance.

When asked, “At what level do you think it is most effective to tackle resistance to antibiotics?”, 43.0% of respondents identified the individual level, specifically targeting prescribers, as the most effective approach. Other responses included environmental/animal health (10.4%), regional/national efforts (12.3%), and global initiatives (10.0%). Additionally, 14.1% of participants emphasized the need for action at all levels, while 10.3% were unsure about the most effective level of intervention.

Regarding strategies employed for prudent antibiotic prescribing, 33.5% recommended using delayed or backup prescribing, 26.6% emphasized patient education, and 21.7% suggested consulting with colleagues. Meanwhile, 12.4% recommended these strategies primarily for new patients, 5.6% did not recommend any specific strategies.

## Bivariable analysis

No statistically significant differences were observed in the "Actual Knowledge" scores across gender (*t* = 2.316, *p* = 0.129), year of study (F = 0.380, *p* = 0.684), or having a family member in the medical field (*t* = 1.837, *p* = 0.176).

Bivariate correlation tests revealed a statistically significant positive correlation between Actual Knowledge and Perceived Knowledge (*r* = 0.205, *p* < 0.05), as well as between Actual Knowledge and Confidence in prescribing antibiotics (*r* = 0.153, *p* < 0.05). Notably, a strong positive correlation was found between Perceived Knowledge and Confidence (*r* = 0.792, *p* < 0.05).

Additionally, the findings demonstrated a positive correlation between Actual Knowledge and attitudes toward the responsibility of combating antibiotic resistance (*r* = 0.192, *p* < 0.05).

A strong positive correlation (*r* = 0.817, *p* < 0.05) was observed between “Confidence” score and “Antibiotic Prescription Guidance Accessibility”. This included access to guidelines, materials for providing advice on prudent antibiotic use, and opportunities to advise individuals on prudent antibiotic use. Students who responded from “strongly disagree” to “strongly agree” reported a lower frequency of antibiotic prescribing.

Further analysis revealed additional significant associations between the current study composite variables and the antibiotic prescribing frequency. Perceived Knowledge (*ρ* = −0.272, *p* < 0.001), Confidence (*ρ* = −0.201, *p* < 0.001), Attitude (*ρ* = −0.258, *p* < 0.001), and Accessibility (*ρ* = −0.205, *p* < 0.001) scores were all significantly and negatively correlated with prescribing frequency, indicating that higher levels of perceived preparedness, access to guidance, and stewardship attitudes were associated with lower reported antibiotic use. In contrast, Actual Knowledge showed no significant association (*ρ* = 0.035, *p* = 0.419). These findings suggest that perceptions, confidence, and system-level support may have a stronger influence on behaviour than factual knowledge alone.

The frequency of antibiotic prescriptions in the last week was significantly associated with the year of study (*χ*^2^ = 55.618, *p* = .000). Advanced students, particularly interns, prescribed antibiotics more frequently than 4th- and 5th-year students.

Knowledge-related variables showed significant negative associations with prescribing frequency. Statements such as “I know what information to give to individuals about prudent use of antibiotics and antibiotic resistance” (*χ*^2^ = 82.650, *p* = .000), “I have sufficient knowledge about how to use antibiotics appropriately for my current training” (*χ*^2^ = 76.644, *p* = .001), and “I am confident in making antibiotic prescribing decisions” (*χ*^2^ = 84.250, *p* = .000) revealed that increased knowledge and confidence correlated with reduced prescribing frequency.

Access to resources also influenced prescribing behaviour. Greater access to guidelines (“I have easy access to guidelines I need on managing infections”, *χ*^2^ = 106.441, *p* = .000) and educational materials “I have easy access to the materials I need to give advice on prudent antibiotic use and antibiotic resistance”, *χ*^2^ = 80.271, *p* = .000) was associated with lower prescribing frequency.

Opportunities to provide advice on prudent antibiotic use were significantly associated with prescribing behaviours (“I have good opportunities to provide advice on prudent antibiotic use to individuals”, *χ*^2^ = 70.586, *p* = .004). Greater opportunities correlated with lower prescribing rates. Confidence in available guidelines (“I have confidence in the antibiotic guidelines available to me”, *χ*^2^ = 79.656, *p* = .000) and feeling supported in decision-making (“I feel supported to not prescribe antibiotics when they are not necessary”, *χ*^2^ = 111.133, *p* = .000) were similarly linked to reduced prescribing frequency.

Considering antibiotic resistance during patient treatment was significantly related to prescribing behaviour (“I consider antibiotic resistance when treating a patient”, *χ*^2^ = 75.524, *p* = .001), with greater consideration associated with lower prescribing frequency.

Bad/inappropriate practices such as prescribing antibiotics due to uncertainty about infection diagnosis (*χ*^2^ = 504.414, *p* = .000), inability to avoid prescribing (*χ*^2^ = 670.491, *p* = .000), and early discontinuation of prescriptions (*χ*^2^ = 609.207, *p* = .000) showed significant positive correlations with prescribing frequency. These challenges increased the frequency of antibiotic prescribing.

When stratified by university, significant associations were observed. Al-Quds University (AQU) students had the highest number of respondents agreeing or strongly agreeing with the following statements:

- “I am confident in making antibiotic prescribing decisions” (*n* = 99, *χ*^2^ = 501.850, *p* = .000).
- “I have a key role in helping control antibiotic resistance” (*n* = 104, *χ*^2^ = 444.476, *p* = .000).
- “I consider antibiotic resistance when treating a patient” (*n* = 110, *χ*^2^ = 35.773, *p* = .008).
- “I have sufficient knowledge about how to use antibiotics appropriately for my current training” (*n* = 105).

A statistically significant association was reported between the statement “I am Confident in Making Antibiotic Prescribing Decisions” with “I have easy access to guidelines I need on managing infections” (*χ*^2^ = 486.332, *p* = .000), “I have access to the materials I need to give advice on prudent antibiotic use and antibiotic resistance” (*χ*^2^ = 555.720, *p* = .000), and “I have good opportunities to provide advice on prudent antibiotic use to individuals” (*χ*^2^ = 703.107, *p* = .000).

Students demonstrated moderate actual knowledge (mean = 4.22/7) and perceived knowledge (mean = 9.45/15). Only 40.3% expressed confidence in their prescribing decisions. Inappropriate prescribing behaviours—such as prescribing due to diagnostic uncertainty (13.3%) and to meet patient expectations (12.1%)—were common. Interns prescribed antibiotics more frequently than 4th- and 5th-year students (*χ*^2^ = 55.618, *p* < 0.001). Access to guidelines and confidence in prescribing were strongly associated with reduced antibiotic use (*p* < 0.001). See Appendix I for detailed results on Tables 2–7.

A strong positive correlation (*r* = 0.817, *p* < 0.05) was observed between the Confidence Composite variable score and the “Antibiotic Prescription Guidance Accessibility” composite variable. The frequency of antibiotic prescriptions during the last week was significantly and positively associated with the year of study (*χ*^2^ = 55.618, *p* = .000), with more advanced students, particularly interns, prescribing antibiotics more frequently compared to earlier-year students (4th- and 5th-years).

Regarding perceived knowledge-related variables and prescribing frequency, a significant negative association was found between the statement “How often did you prescribe antibiotics during the last one week?” and each of the following:

- “I know what information to give to individuals about prudent use of antibiotics and antibiotic resistance” (*χ*^2^ = 82.650, *p* = .000).
- “I have sufficient knowledge about how to use antibiotics appropriately for my current training” (*χ*^2^ = 76.644, *p* = .001).
- “I am confident in making antibiotic prescribing decisions” (*χ*^2^ = 84.250, *p* = .000).

In each case, increased knowledge and confidence were associated with a lower frequency of antibiotic prescribing.

Access to resources showed significant associations with prescribing behaviour. Greater access to guidelines, “I have easy access to the guidelines I need on managing infections”, (*χ*^2^ = 106.441, *p* = .000) and educational materials “I have easy access to the materials I need to give advice on prudent antibiotic use and antibiotic resistance”, (*χ*^2^ = 80.271, *p* = .000) was associated with a lower frequency of antibiotic prescribing.

Opportunities to provide advice on antibiotic use were also significantly associated with prescribing behaviours, as reflected in responses to “I have good opportunities to provide advice on prudent antibiotic use to individuals” (*χ*^2^ = 70.586, *p* = .004), where better opportunities correlated with lower prescribing rates. Confidence in available guidelines (“I have confidence in the antibiotic guidelines available to me”, (*χ*^2^ = 79.656, *p* = .000) and feeling supported in decision-making (“I feel supported to not prescribe antibiotics when they are not necessary”, *χ*^2^ = 111.133, *p* = .000) were similarly associated with a lower frequency of prescribing.

Considering antibiotic resistance during patient treatment was also significantly related to prescribing behaviours “I consider antibiotic resistance when treating a patient”, (*χ*^2^ = 75.524, *p* = .001), Students who responded from “strongly disagree” to “strongly agree” with the previous statement reported a lower frequency of antibiotic prescribing.

Higher prescription frequency was correlated positively with some inappropriate practice variables, such as “How often would you have preferred not to prescribe an antibiotic but were not able during the last one week?” (*χ*^2^ = 670.491, *p* = .000), “How often did you stop an antibiotic prescription earlier than the prescribed course length during the last one week?” (*χ*^2^ = 609.207, *p* = .000), and “How often did you prescribe an antibiotic because you were uncertain about the diagnosis of infection during the last one week?” (*χ*^2^ = 504.414, *p* = .000).

When stratified by university, significant associations were found for several statements. For instance, Al-Quds University (AQU) had the highest number of students who agreed or strongly agreed with the statement “I am confident in making antibiotic prescribing decisions” (*n* = 99, *χ*^2^ = 501.850, *p* = .000). Similarly, AQU students also reported the highest number agreeing with the statement “I have a key role in helping control antibiotic resistance” (*n* = 104, *χ*^2^ = 444.476, *p* = .000). Regarding the statement “I consider antibiotic resistance when treating a patient” (*χ*^2^ = 35.773, *p* = .008), AQU again had the highest number of students who agreed or strongly agreed (*n* = 110), followed by Arab American University (AAU) (*n* = 68). Furthermore, for the statement “I have sufficient knowledge about how to use antibiotics appropriately for my current training”, AQU students were again the most likely to agree or strongly agree (*n* = 105, *χ*^2^ = 42.132, *p* = .000). Table 8A presents the abbreviated bivariate analysis results. See Appendix I, Table 8B for complete bivariate analysis results.

**Table 8A:** Bivariate analysis (Chi-square tests, *P*-value).

Variable	*χ*² Value	*P*-value
Year of study vs. prescribing frequency	55.618	<0.001
Confidence vs. prescribing frequency	84.250	<0.001
Access to guidelines vs. precribing frequency	106.441	<0.001
Bad/inappropriate practices vs. prescribing frequency	670.491	<0.001

**Table 8B:** Bivariate analysis (Chi-square tests, *P*-value) between study main variables.

Study variables		Chi-Square test value	Sig. (2-tailed)*
			“*p*-Value”
The frequency of antibiotic prescriptions within the last week (How often did you prescribe antibiotics during the last one week?)	Year of study	55.618	.000
	I know what information to give to individuals about prudent use of antibiotics and antibiotic resistance	82.650	.000
	I have sufficient knowledge about how to use antibiotics appropriately for my current training	76.644	.001
	I have a key role in helping control antibiotic resistance	64.037	.016
	I have easy access to guidelines I need on managing infections	106.441	.000
	I have easy access to the materials I need to give advice on prudent antibiotic use and antibiotic resistance	80.271	.000
	I have good opportunities to provide advice on prudent antibiotic use to individuals	70.586	.004
	I am confident in making antibiotic prescribing decisions	84.250	.000
	I have confidence in the antibiotic guidelines available to me	79.656	.000
	I consider antibiotic resistance when treating a patient	75.524	.001
	I feel supported to not prescribe antibiotics when they are not necessary	111.133	.000
	How often would you have preferred not to prescribe an antibiotic but were not able during the last one week?	670.491	.000
	How often did you stop an antibiotic prescription earlier than the prescribed course length during the last one week?	609.207	.000
	How often did you prescribe an antibiotic because you were uncertain about the diagnosis of infection during the last one week?	504.414	.000
I have sufficient knowledge about how to use antibiotics appropriately for my current training	University	42.132	.001
	I know what information to give to individuals about prudent use of antibiotics and antibiotic resistance	561.601	.000
	I have a key role in helping control antibiotic resistance	444.476	.000
	I have easy access to guidelines I need on managing infections	457.935	.000
	I have easy access to the materials I need to give advice on prudent antibiotic use and antibiotic resistance	460.337	.000
	I have good opportunities to provide advice on prudent antibiotic use to individuals	487.876	.000
	I am confident in making antibiotic prescribing decisions	501.850	.000
	I have confidence in the antibiotic guidelines available to me	463.639	.000
	I consider antibiotic resistance when treating a patient	395.389	.000
	I feel supported to not prescribe antibiotics when they are not necessary	339.789	.000
	How often would you have preferred not to prescribe an antibiotic but were not able during the last one week?	132.679	.000
	How often did you stop an antibiotic prescription earlier than the prescribed course length during the last one week?	114.887	.000
	How often did you prescribe an antibiotic because you were uncertain about the diagnosis of infection during the last one week?	113.470	.000
I feel supported to not prescribe antibiotics when they are not necessary	I know what information to give to individuals about prudent use of antibiotics and antibiotic resistance	396.825	.000
	I have a key role in helping control antibiotic resistance	327.801	.000
	I have easy access to guidelines I need on managing infections	364.558	.000
	I have easy access to the materials I need to give advice on prudent antibiotic use and antibiotic resistance	334.137	.000
	I have good opportunities to provide advice on prudent antibiotic use to individuals	480.258	.000
	I am confident in making antibiotic prescribing decisions	574.228	.000
	I have confidence in the antibiotic guidelines available to me	534.556	.000
	I consider antibiotic resistance when treating a patient	792.468	.000
	How often would you have preferred not to prescribe an antibiotic but were not able during the last one week?	123.911	.000
	How often did you stop an antibiotic prescription earlier than the prescribed course length during the last one week?	168.159	.000
	How often did you prescribe an antibiotic because you were uncertain about the diagnosis of infection during the last one week?	131.618	.000
	I have sufficient knowledge about how to use antibiotics appropriately for my current training	339.789	.000
I have a key role in helping control antibiotic resistance	University	32.324	.020
	I know what information to give to individuals about prudent use of antibiotics and antibiotic resistance	475.464	.000
	I have easy access to guidelines I need on managing infections	440.596	.000
	I have easy access to the materials I need to give advice on prudent antibiotic use and antibiotic resistance	436.202	.000
	I have good opportunities to provide advice on prudent antibiotic use to individuals	404.177	.000
	I am confident in making antibiotic prescribing decisions	428.367	.000
	I have confidence in the antibiotic guidelines available to me	393.710	.000
	I consider antibiotic resistance when treating a patient	371.923	.000
	How often would you have preferred not to prescribe an antibiotic but were not able during the last one week?	105.887	.000
	How often did you stop an antibiotic prescription earlier than the prescribed course length during the last one week?	121.877	.000
	How often did you prescribe an antibiotic because you were uncertain about the diagnosis of infection during the last one week?	89.318	.000
	I have sufficient knowledge about how to use antibiotics appropriately for my current training	444.476	.000
	I feel supported to not prescribe antibiotics when they are not necessary	327.801	.000
I am confident in making antibiotic prescribing decisions	I know what information to give to individuals about prudent use of antibiotics and antibiotic resistance	488.086	.000
	I have easy access to guidelines I need on managing infections	486.332	.000
	I have easy access to the materials I need to give advice on prudent antibiotic use and antibiotic resistance	555.720	.000
	I have good opportunities to provide advice on prudent antibiotic use to individuals	703.107	.000
	I have confidence in the antibiotic guidelines available to me	937.561	.000
	I consider antibiotic resistance when treating a patient	597.429	.000
	How often would you have preferred not to prescribe an antibiotic but were not able during the last one week?	120.032	.000
	How often did you stop an antibiotic prescription earlier than the prescribed course length during the last one week?	131.451	.000
	How often did you prescribe an antibiotic because you were uncertain about the diagnosis of infection during the last one week?	127.747	.000
	I have sufficient knowledge about how to use antibiotics appropriately for my current training	501.850	.000
	I feel supported to not prescribe antibiotics when they are not necessary	574.228	.000
	I have a key role in helping control antibiotic resistance	428.367	.000
I consider antibiotic resistance when treating a patient	University	35.773	.008
	I know what information to give to individuals about prudent use of antibiotics and antibiotic resistance	425.149	.000
	I have easy access to guidelines I need on managing infections	379.562	.000
	I have easy access to the materials I need to give advice on prudent antibiotic use and antibiotic resistance	403.685	.000
	I have good opportunities to provide advice on prudent antibiotic use to individuals	445.735	.000
	I have confidence in the antibiotic guidelines available to me	662.110	.000
	How often would you have preferred not to prescribe an antibiotic but were not able during the last one week?	114.799	.000
	How often did you stop an antibiotic prescription earlier than the prescribed course length during the last one week?	120.745	.000
	How often did you prescribe an antibiotic because you were uncertain about the diagnosis of infection during the last one week?	142.039	.000
	I have sufficient knowledge about how to use antibiotics appropriately for my current training	395.389	.000
	I feel supported to not prescribe antibiotics when they are not necessary	792.468	.000
	I have a key role in helping control antibiotic resistance	371.923	.000
	I am confident in making antibiotic prescribing decisions	597.429	.000

* means ≤ 0.05.

## Discussion

Antibiotic resistance is a global health crisis, exacerbated by the overuse and misuse of antibiotics across various healthcare sectors, including dentistry. While existing studies have explored antibiotic knowledge and prescribing behaviours among practicing dentists, limited attention has been given to dental students, particularly in Palestine. This study is the first of its kind in Palestine, addressing a critical gap in the literature regarding dental students' knowledge and practices related to antibiotic prescriptions.

The findings reveal that while Palestinian dental students possess foundational knowledge about antibiotic resistance, significant gaps exist in their confidence and ability to apply this knowledge in clinical practice. A statistically significant positive correlation was observed between actual knowledge and confidence in prescribing practices. However, a substantial proportion of students reported inappropriate prescribing behaviours, often driven by uncertainty in diagnosis, fear of complications, time constraints, and patient expectations.

Our findings align with existing literature, highlighting significant discrepancies between knowledge and clinical practices in antibiotic prescribing among dental practitioners globally, including those in the Middle East. While dentists generally demonstrate a strong theoretical understanding of antibiotic use, studies consistently report widespread inappropriate prescribing, contributing to the growing challenge of antimicrobial resistance.

For instance, studies in Croatia, Bosnia, and Serbia found that dentists achieved high antibiotic resistance knowledge scores (6.40 out of 8); however, many still prescribed antibiotics at patient request, reflecting the influence of patient expectations on prescribing behaviours (20). Similarly, in Egypt, dental students frequently overprescribed antibiotics to satisfy patient demands (21). Roganović and Barac (22) further emphasized that inadequate ethical training contributed to inappropriate prescribing among European dental students, a concern echoed in our study. These ethical dilemmas highlight the need for enhanced training that emphasizes both evidence-based decision-making and ethical prescribing practices.

A concerning trend emerged among senior students, particularly interns, who exhibited a higher frequency of antibiotic prescriptions compared to their junior counterparts, despite having similar levels of theoretical knowledge. This aligns with findings from Jordan, where majority of dentists understood resistance mechanisms but lacked confidence in their prescribing decisions (18). Similarly, Palestinian students faced challenges akin to those identified in the Asia-Pacific region, where awareness of antimicrobial resistance did not translate into confident prescribing decisions (23). In contrast, Saudi dental students demonstrated overconfidence with increasing experience, leading to higher prescribing rates (24). These trends suggest that clinical exposure alone does not necessarily improve antibiotic stewardship and may, in some cases, reinforce inappropriate prescribing habits. The need for clear guidelines for both dental students and dentists is critical to promote appropriate antibiotic prescription practices.

In the current study, the absence of standardized clinical guidelines exacerbated inappropriate prescribing. A study among UK dentists demonstrated that guideline adherence improved prescribing appropriateness by 30% (5), reinforcing the importance of clear, standardized protocols. Consistently, our study found a statistically significant association between access to guidelines and lower prescribing frequency, underscoring the need for national and institutional policies to regulate antibiotic use.

A key finding of this study is the persistent gap between theoretical knowledge and practical application, mirroring trends observed in other regions. Tariq et al. (25) reported that although Brazilian dental students possessed general awareness of antibiotic resistance, a substantial gap remained in translating this knowledge into appropriate clinical decision-making. Similarly, a study in the United Arab Emirates highlighted moderate knowledge of antibiotics but significant misconceptions regarding their appropriate use and resistance mechanisms (26). These misconceptions reflect broader deficiencies in dental education that may negatively impact future prescribing behaviours. One potential contributor to these gaps is inadequate interprofessional collaboration and limited training with other healthcare professionals, which may hinder effective coordination in antibiotic stewardship efforts (27).

A European study identified seven key factors driving inappropriate prescribing, including reliance on outdated educational materials and inconsistencies in guidelines (28). Likewise, Suda et al. found that US dentists exhibited inappropriate prophylaxis prescribing in 80% of cases, often prioritizing perceived patient needs over established guidelines (29). These global patterns highlight the urgent need for curricular reforms that integrate evidence-based prescribing guidelines and interdisciplinary collaboration into dental education.

Despite widespread awareness of antibiotic resistance, the persistent gap between knowledge and practice underscores the need for continuous education and intervention strategies. Our study contributes to global antibiotic stewardship efforts by identifying key predictors of prescribing behaviours, including perceived knowledge, actual knowledge, attitudes, and confidence. Importantly, the use of composite scores in our analysis revealed that perceived knowledge, confidence, attitude toward stewardship, and access to prescribing resources were all significantly and negatively associated with antibiotic prescribing frequency. These findings indicate that improving students' self-efficacy, motivation, and access to support tools may be as important as increasing factual knowledge in reducing unnecessary prescribing. Interestingly, actual knowledge did not show a significant association with prescribing frequency, highlighting a misalignment between what students know and how they act. This reinforces the importance of educational interventions that go beyond knowledge transmission and focus on behavioral, environmental, and structural drivers of prescribing habits.

Furthermore, our study evaluated whether student responses to key stewardship questions deviated significantly from expected distributions. The results supported the need for targeted improvements in areas such as confidence and resource accessibility, where observed response patterns were skewed toward uncertainty or lack of access. These findings provide concrete areas for educational and policy-based interventions.

Addressing these factors through structured educational interventions, standardized guidelines, and policy-driven reforms could significantly enhance prescribing confidence and promote responsible antibiotic use among future dental practitioners.

A recent systematic review found that dentists contribute to approximately 10% of all antibiotic prescriptions globally, with up to 90% of these being inappropriate (30). This reinforces the urgent need for enhanced antibiotic stewardship programs tailored to both practicing dentists and dental students. Strengthening appropriate prescribing behaviors requires a multi-faceted approach that integrates education, policy, and institutional support. Implementing evidence-based guidelines, improving access to up-to-date prescribing protocols, and fostering interprofessional collaboration could bridge the gap between knowledge and clinical practice, ultimately mitigating the impact of antibiotic resistance on global public health.

Aligning with global strategies, standardized guidelines, audit-feedback mechanisms, and stewardship training are pivotal in combating antimicrobial resistance (AMR). The WHO Global Action Plan (31) prioritizes education and the implementation of guidelines, reinforcing the need for structured stewardship programs in dental education. A successful model can be drawn from UK dental schools, where mandatory stewardship modules significantly reduced inappropriate prescribing, suggesting a viable framework for Palestinian dental education (32).

The findings of this study highlight the urgent need to integrate structured antibiotic stewardship programs into dental curricula. By providing students with access to updated clinical guidelines and organizing targeted training workshops on prudent antibiotic use, dental institutions can strengthen evidence-based prescribing practices and bridge the gap between theoretical knowledge and clinical decision-making. Additionally, standardized guidelines for antibiotic prescribing in dentistry, aligned with the best global practices, should be established to ensure consistency in prescribing behaviours.

From a policy perspective, effective interventions such as audit and feedback mechanisms have demonstrated potential in improving prescribing behaviours, though further research is needed to assess their long-term impact (30). Educational initiatives that address modifiable factors—such as prescribing confidence, ethical decision-making, and reliance on updated materials—are essential to curbing inappropriate antibiotic use (33). By integrating these strategies into dental education and healthcare policy, institutions can play a crucial role in mitigating antibiotic resistance and fostering responsible prescribing practices among future dental professionals.

Despite its contributions, this study has certain limitations. First, the cross-sectional design captures only a snapshot of students' knowledge and behaviours, limiting the ability to assess changes over time. Additionally, self-reported data may introduce response bias, as students might overestimate their confidence or underreport inappropriate practices. The study was also confined to Palestinian dental schools, and while it provides valuable regional insights, findings may not be directly generalizable to other populations. Future longitudinal studies are needed to assess how educational interventions influence prescribing behaviours over time.

Future research should explore the long-term impact of structured antibiotic stewardship training on students' prescribing behaviours. Additionally, qualitative studies could provide deeper insights into the barriers dental students face in adhering to antibiotic guidelines.

Expanding this research to practicing dentists and comparing their prescribing patterns with those of students would also offer valuable perspectives on how knowledge translates into professional practice.

## Conclusion

This study identifies some important gaps in the knowledge, confidence, and prescribing behaviours of Palestinian dental students with regard to antibiotic stewardship. The findings underscore a pressing need for tailored educational interventions, enhanced access to prescribing guidelines, and the introduction of policy-driven initiatives to encourage more responsible antibiotic use in dental practice. Addressing these gaps is crucial to mitigating the rise of antibiotic resistance and fostering prudent prescribing practices among future dental professionals.

The insights gained from this study are particularly valuable for key stakeholders, including policymakers, dental educators, professional associations, and the Ministry of Health. These groups can leverage the findings to design targeted educational programs and update guidelines on optimal antibiotic prescribing in dentistry. As the first study of its kind in Palestine, this research establishes a vital baseline that can inform future policy development and educational strategies, contributing to global efforts to safeguard antibiotic efficacy and improve patient outcomes.

While acknowledging the study's limitations, it is essential that future research investigates the long-term impact of specific educational interventions. Expanding the study to broader geographical regions would further enrich our understanding. By addressing these gaps, we can better equip the next generation of dental professionals to tackle the global challenge of antibiotic resistance, ensuring they are well-prepared to contribute effectively to public health initiatives and provide responsible, evidence-based patient care.

## Data Availability

The raw data supporting the conclusions of this article will be made available by the authors, without undue reservation.
